# Prognostic importance of apathy in syndromes associated with frontotemporal lobar degeneration

**DOI:** 10.1212/WNL.0000000000007249

**Published:** 2019-04-02

**Authors:** Claire J. Lansdall, Ian T.S. Coyle-Gilchrist, Patricia Vázquez Rodríguez, Alicia Wilcox, Eileen Wehmann, Trevor W. Robbins, James B. Rowe

**Affiliations:** From the Departments of Clinical Neurosciences (C.J.L., I.T.S.C.-G., P.V.R., A.W., E.W., J.B.R.) and Psychology (T.W.R.), and Behavioral and Clinical Neuroscience Institute (T.W.R., J.B.R.), University of Cambridge, UK; University Medical Centre Hamburg-Eppendorf (E.W.), University of Hamburg, Germany; and MRC Cognition and Brain Sciences Unit (J.B.R.), Cambridge, UK.

## Abstract

**Objective:**

To determine the influence of apathy, impulsivity, and behavioral change on survival in patients with frontotemporal dementia, progressive supranuclear palsy, and corticobasal syndrome.

**Methods:**

We assessed 124 patients from the epidemiologic PiPPIN (Pick's Disease and Progressive Supranuclear Palsy, Prevalence and Incidence) study. Patients underwent detailed baseline cognitive and behavioral assessment focusing on apathy, impulsivity, and behavioral change. Logistic regression identified predictors of death within 2.5 years from assessment, including age, sex, diagnosis, cognition, and 8 neurobehavioral profiles derived from a principal component analysis of neuropsychological and behavioral measures.

**Results:**

An apathetic neurobehavioral profile predicted death (Wald statistic = 8.119, *p* = 0.004, Exp(B) = 2.912, confidence interval = >1 [1.396–6.075]) and was elevated in all patient groups. This profile represented apathy, weighted strongly to carer reports from the Apathy Evaluation Scale, Neuropsychiatric Inventory, and Cambridge Behavioral Inventory. Age at assessment, sex, and global cognitive impairment were not significant predictors. Differences in mortality risk across diagnostic groups were accounted for by their neuropsychiatric and behavioral features.

**Conclusions:**

The relationship between apathy and survival highlights the need to develop more effective and targeted measurement tools to improve its recognition and facilitate treatment. The prognostic importance of apathy suggests that neurobehavioral features might be useful to predict survival and stratify patients for interventional trials. Effective symptomatic interventions targeting the neurobiology of apathy might ultimately also improve prognosis.

Dementia greatly increases mortality but the mechanisms of this effect are poorly understood. In the clinical syndromes associated with frontotemporal lobar degeneration (FTLD), there is substantial variation in survival rates. For example, survival is shortest for frontotemporal dementia (FTD) with motor neuron disease (typically 2–5 years), intermediate for progressive supranuclear palsy (PSP [Richardson syndrome]; typically 5–7 years),^1,2^ corticobasal syndrome (CBS; typically 6–8 years),^3^ and behavioral variant FTD (bvFTD; typically 5–8 years),^2^ and longest in semantic variant primary progressive aphasia (svPPA; typically 10–12 years)^2,3^ or PSP-parkinsonism (typically 10–12 years).^4^ Despite improved knowledge of the clinical features and neuropathologic hallmarks of these FTLD syndromes, their influence on survival is unclear.

Predicting disease progression and patient trajectories is challenging, and the mechanisms underlying variations in survival rates remain elusive. Previous studies have reported only mild or null associations between survival and patient demographics, a positive family history, and dementia severity at the time of diagnosis, while highlighting the importance of neuropsychiatric and behavioral features.^3,5,6^ However, variations in estimated survival rates in part reflect the use of clinical^3,7^ vs neuropathologic^5,8^ cohorts. Limited clinicopathologic correlations and inclusion of nonprogressive “phenocopy” patients may have affected previous estimates. Indeed, the removal of 24 such “phenocopy” cases from 91 clinical bvFTD cases reduced median survival from 9.0 to 7.6 years from onset.^7^

Improved clinical diagnostic criteria^9^ and assessment of the “spectrum” of FTLD^10^ present new opportunities to understand the determinants of poor survival, whether for disorders associated with primary tauopathies (PSP, CBS, nonfluent variant PPA, and half of bvFTD) or TDP-43 (TAR DNA-binding protein 43) pathologies (svPPA and half of bvFTD). Herein, we focus on the neuropsychiatric features that arise in many disorders of movement and cognition. For example, apathy has been associated with worse outcomes across a range of neurologic conditions including Alzheimer disease, stroke,^11^ Huntington disease,^12^ Parkinson disease,^13^ FTLD syndromes,^14–16^ and predementia states.^17^ Apathy correlates with poor functioning, caregiver distress,^15^ cognitive decline,^13^ increased dementia conversion rates, reduced quality of life,^11^ and poor prognosis.^18^ The association between apathy and survival may be causal, or reflect common influences of a third factor on both and disease-specific factors. However, a direct influence of apathy on survival warrants further investigation, not least because effective symptomatic treatments might also indirectly improve prognosis.^6,19,20^

We used the Pick's Disease and Progressive Supranuclear Palsy Prevalence and Incidence (PiPPIN)^2^ cohort to test the hypothesis that neurobehavioral components of FTLD syndromes are significant predictors of mortality. We used logistic regression to estimate the probability of death occurring within 2.5 years from assessment. Candidate predictor variables included age, sex, cognitive status, diagnosis, and the 8 neuropsychological principal components reported in reference 10. In view of previous studies highlighting the importance of apathy and related behavioral change over demographics, diagnosis, and cognitive status, we hypothesized that the neurobehavioral components strongly weighted toward assessment of apathy would be most influential on survival. Specifically, we predicted that carer-based estimates of apathy, everyday skills, and challenging behaviors would predict mortality, across the spectrum of disorders caused by FTLD.

## Methods

### Standard protocol approvals, registrations, and patient consents

The study was approved by the Cambridge 2 research ethics committee (reference 12/EE/0475) and supported by the National Institute for Health Research clinical research network (ID-15504). Informed consent was obtained at each study visit, with the personal consultee process used for participants who lacked mental capacity, in accordance with UK law. We anticipated approximately 150 patients based on prior epidemiologic estimates, which would provide good power (>0.8) to detect small- to medium-sized group effects and correlations, and to identify >4 distinct neuropsychological components.

### Cohort

Two hundred four patients were identified and recruited to the PiPPIN study, according to consensus clinical diagnostic criteria,^2^ plus 50 healthy age- and sex-matched controls. The occurrence and date of death were obtained from centralized United Kingdom National Health Service records. Exclusions for the current analysis included insufficient complete data for logistic regression (e.g., limited cognitive/functional, self-rated, carer-rated, or behavioral assessment), or assessment in the PiPPIN study too late for the follow-up interval at the time of analysis. Because logistic regression removes cases with missing data “list-wise” by default, the inclusion of multiple predictor variables (each of which may have a small but finite percentage of missing data) results in the additional exclusion of patients. Of the 204 PiPPIN patients, 124 undertook detailed neuropsychological assessments (PSP 35, CBS 29, PPA 33, bvFTD 27), of whom 112 had at least 30 months of follow-up at the time of analysis. The 50 healthy controls also undertook the neuropsychological assessment.

Participants were tested while on their usual medication: 40% took “antidepressant” medications (for affective or behavioral indications), 29% dopaminergic medication, 4% antipsychotic medication, and 37% other centrally acting medications (benzodiazepines, antiepileptic, analgesics, pregabalin, or cholinesterase inhibitors).

### Statistical analysis

All statistical analyses were performed using SPSS Statistics version 22 (IBM Corp., Armonk, NY). Kaplan-Meier survival curves were used to illustrate survival from assessment date and from estimated onset, by diagnostic group. Survival rates were compared by χ^2^ test. Logistic regression was used to identify significant predictors of death within 30 months from assessment. The principal outcome measure refers to 30 months because of similar sample sizes across groups (deceased = 50 and alive = 62). To predict survival at 30 months, patients were classified as “deceased” or “alive” using a cutoff of 913 days post assessment. Patients who were alive but had not yet lived 30 months from assessment were classified as “insufficient follow-up time” and excluded.

Logistic regression used the “Enter” method. Predictor variables included age at assessment, sex, cognitive status (Addenbrooke's Cognitive Examination–Revised),^21^ diagnosis (PSP, CBS, PPA, bvFTD), and the 8 principal behavioral components extracted from a neuropsychological and behavioral test battery of participants, and questionnaire responses of carers and clinician, using the principal components method.^10^ In brief, 22 questionnaires and behavioral measures assessing apathy, impulsivity, and related behavioral change were obtained, gaining insight from multiple perspectives including patient, carer, clinician, and objective tasks. The neurocognitive components revealed that (1) apathy and impulsivity are *positively* correlated, with validated measures of each loading onto the same components; (2) these behaviors are present in all syndromes associated with FTLD, to a varying degree; and (3) patient, carer, and objective measures differ, loading onto separable components and reflecting distinct neural correlates.^10^

Overall fit of the model was determined by the −2 log-likelihood and its associated χ^2^ statistic, using a threshold of *p* < 0.05 indicating a significant fit of the data. Cox and Snell *R*^2^ values provided an additional indication of model effect size. The influence of the independent variables on predicting outcome (death within 2.5 years) were determined by the significance of the Wald statistic (*p* < 0.05). Additional information regarding the directionality of effect was provided by the odds ratio [(Exp(B)]; values >1 indicate increasing odds of outcome occurrence (death) with increased values of the predictor variable, while values <1 indicate decreasing odds of outcome occurrence with increased values of the predictor variable). Confidence intervals of the Exp(B) values were used to confirm the direction of the relationship in the population. Classification accuracy of the final model was compared to the baseline model (baseline model [constant only] % − new model [all predictor variables] %) to determine whether inclusion of the independent variables resulted in significant model improvement. Residuals were also examined to assess model fit. Statistics included the following: standardized residuals to measure the model fit to the sample data (<1% of observations ±2.58); Cook distance to measure the overall influence of an individual case on the model (values <1); and DFBeta statistics to measure the influence of a case on the values of *b* (values <1).

Sensitivity (the percentage of cases that have the observed characteristic and were correctly predicted by the model [true positives]) and specificity (the percentage of cases that did not have the observed characteristics and were correctly predicted as not having it [true negatives]) of the model were also calculated (positive predictive value = number of true positives/[number of true positives + number of false positives]; negative predictive value = number of true negatives/[number of true negatives + number of false negatives]).

### Data availability

Anonymized data may be shared by request from a qualified investigator for noncommercial purposes, subject to participants' prior consent to data sharing.

## Results

### Cohort, demographics, and clinical features

Demographics and clinical features of the 124 patients and 50 healthy controls at baseline are detailed in table 1. The patients included in the logistic regression analysis were similar to those who were not included, in terms of age, sex, and diagnostic group (table 2). The neurobehavioral profiles are summarized in table 3, in terms of the loadings of each test in a principal component. The correlations of components with measures of cognition and function are reported in table 4.

**Table 1:**
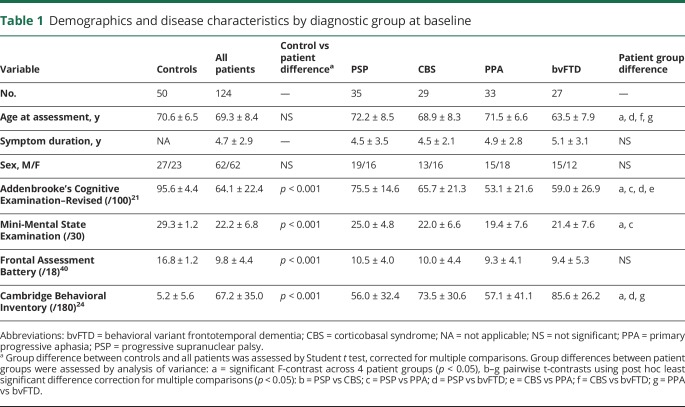
Demographics and disease characteristics by diagnostic group at baseline

**Table 2:**
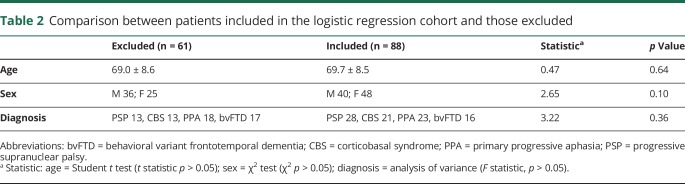
Comparison between patients included in the logistic regression cohort and those excluded

**Table 3:**
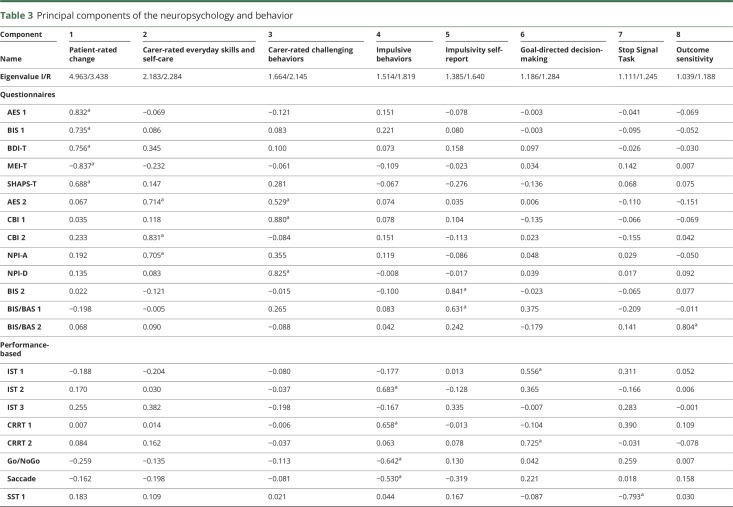

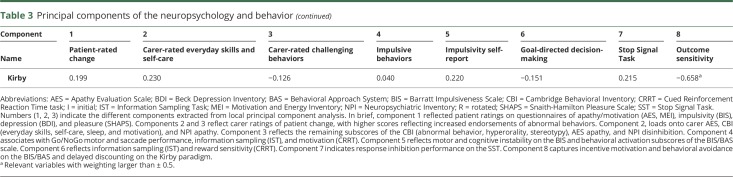
Principal components of the neuropsychology and behavior

**Table 4:**
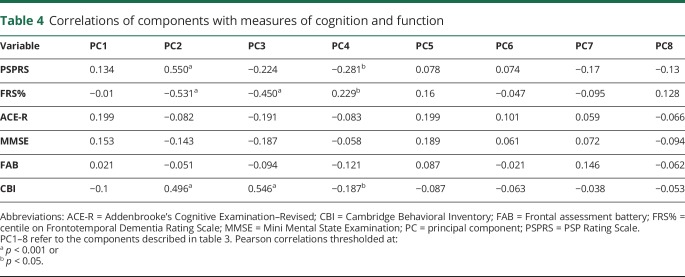
Correlations of components with measures of cognition and function

At 30 months post assessment, 50 patients had died, including 21 PSP, 15 CBS, 5 PPA, and 9 bvFTD. The remaining patients were classified as “alive” (n = 62) or were excluded because of insufficient follow-up time (n = 12). Twenty-four cases were removed list-wise during the logistic regression because of missing data of interest. The final logistic regression subset was representative of the full cohort from which the neurobehavioral profiles were derived,^10^ in terms of age, sex, and diagnosis (table 2).

Survival ranged from 22 to 910 days after assessment (PSP 64–881 days, CBS 22–791 days, PPA 308–910 days, bvFTD 261–761 days). Of the 124 patients, 42 had died at 24 months, including 20 PSP, 11 CBS, 3 PPA, and 8 bvFTD, 81 were alive, and one had insufficient follow-up time. At 36 months, 55 patients had died including 22 PSP, 18 CBS, 6 PPA, and 9 bvFTD, 39 were alive, and 30 had insufficient follow-up time.

### Logistic regression

Including all predictors resulted in a significant fit to the model (−2 log likelihood = 88.401, χ^2^ = 29.9, degrees of freedom [*df*] = 14, *p* = 0.008, Cox and Snell *R*^2^ = 0.288, Nagelkerke *R*^2^ = 0.390). Model classification accuracy improved from 60.2% at baseline (including only a constant) to 73.9% following inclusion of the predictor variables. The model correctly classified 43 as alive while incorrectly classifying 13, and correctly classified 22 as dead while incorrectly classifying an additional 10, resulting in a positive predictive value of 81% and negative predictive value of 63% (positive predictive value = 43/43 + 10 = 0.811, negative predictive value = 22/22 + 13 = 0.629).

**Figure 1:**
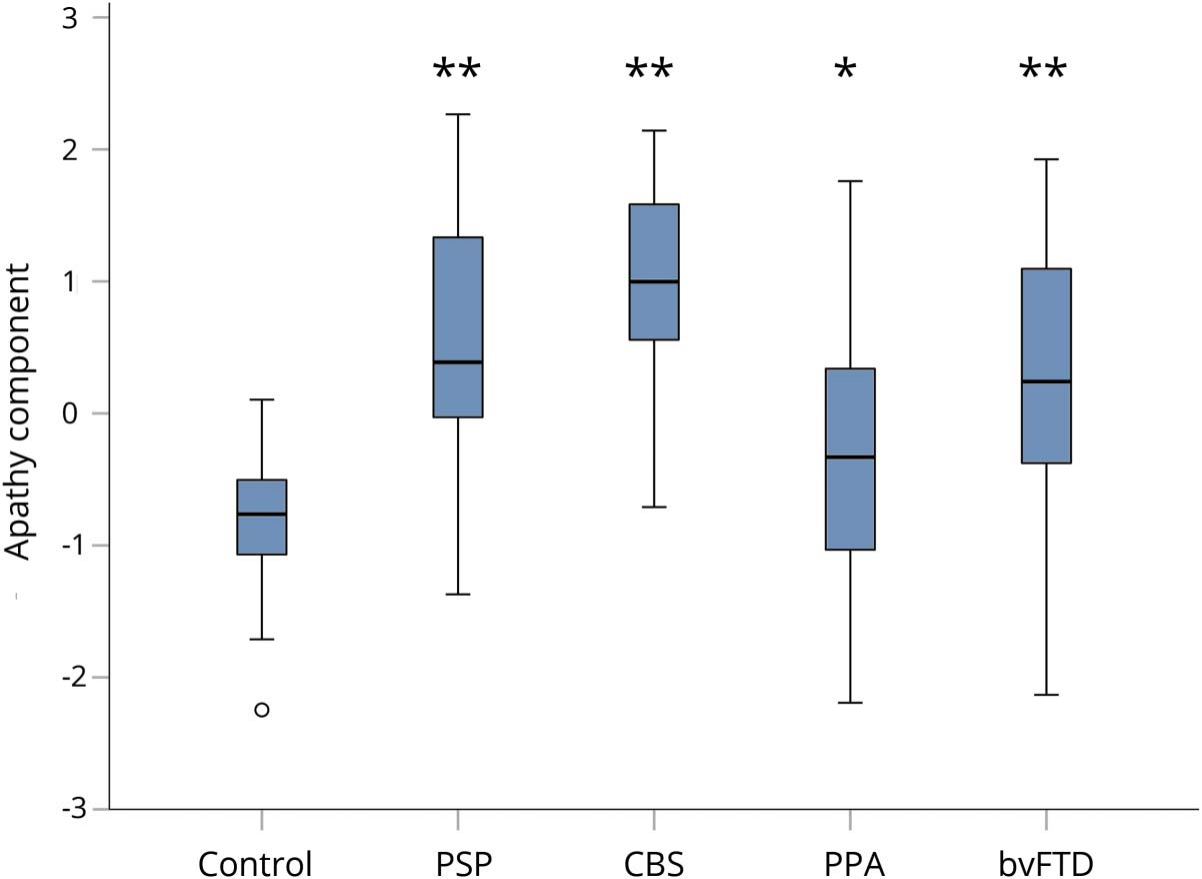
Boxplot of apathy (component 2) by diagnostic group Patients scored significantly higher than controls on component 2 across diagnostic groups (analysis of variance, least significant difference correction for multiple comparisons, control vs PSP, CBS, bvFTD **p < 0.001, vs PPA *p < 0.05). Component 2 had strong loadings from the carer-rated Apathy Evaluation Scale, Neuropsychiatric Inventory apathy subscore, and Cambridge Behavioral Inventory subscores of everyday skills, self-care, sleep, and motivation. Overall, higher scores on this neurobehavioral component reflected increased endorsement of apathy. bvFTD = behavioral variant frontotemporal dementia; CBS = corticobasal syndrome; PPA = primary progressive aphasia; PSP = progressive supranuclear palsy.

Of the predictor variables, carer-rated change in everyday skills, self-care, and apathy (component 2) were the most significant predictor of death within 2.5 years from PiPPIN assessment (Wald statistic = 8.119, *p* = 0.004, Exp(B) *=* 2.912, confidence interval = >1 [1.396–6.075]; table 5). An Exp(B) value >1 (and confidence intervals both >1) indicated that increases in component 2 (weighted toward the carer-rated Apathy Evaluation Scale,^22^ Neuropsychiatric Inventory apathy subscore,^23^ and Cambridge Behavioral Inventory^24^ subscores of everyday skills, self-care, sleep, and motivation) significantly increased the odds of death within the 2.5-year time period. All patient groups scored significantly higher than controls on component 2 (figure 1).

**Table 5:**
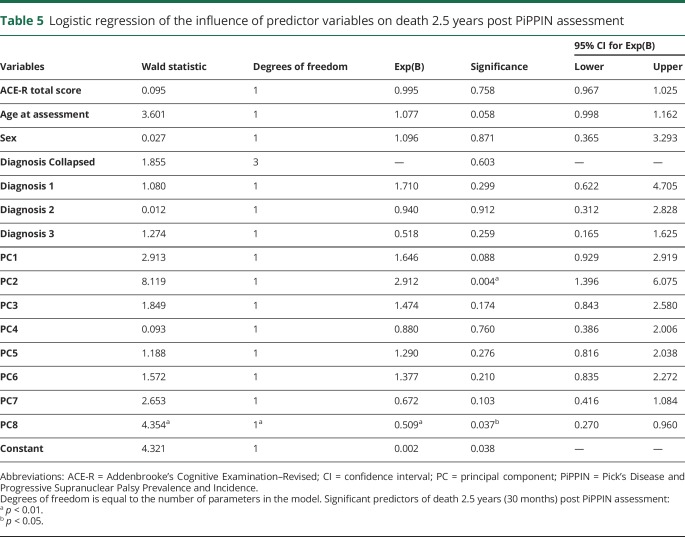
Logistic regression of the influence of predictor variables on death 2.5 years post PiPPIN assessment

Component 8, reflecting insensitivity to reward on the Kirby and behavioral inhibition on the Barratt Impulsiveness Scale/Behavioral Activation System, was marginal. The effect of age at assessment did not reach significance. Examination of the residuals confirmed good model fit: standardized residuals were all within ±2.58, Cook distance, and DFBeta values were <1.

### Kaplan-Meier survival analysis

**Figure 2:**
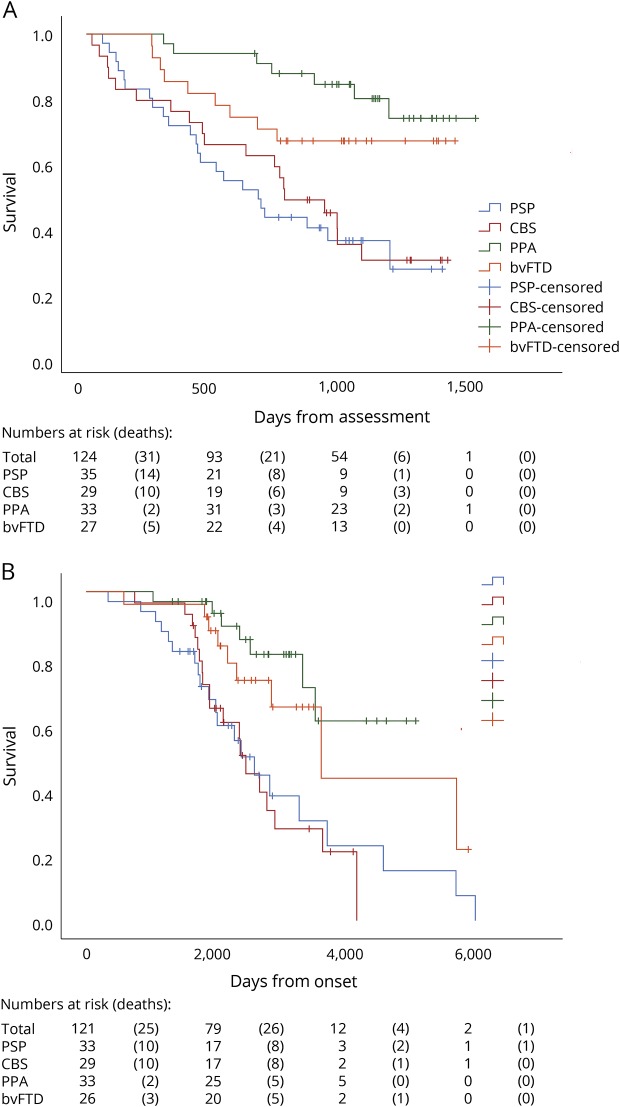
Kaplan-Meier survival curves showing days since assessment and days since reported symptom onset Survival rates differed significantly between diagnostic groups from PiPPIN assessment (A: log-rank [Mantel-Cox] χ^2^ = 20.9, *df* = 3, *p* < 0.001; pairwise comparisons revealed significant differences for PSP vs PPA χ^2^ = 17.0, *p* < 0.001; PSP vs bvFTD χ^2^ = 6.0, *p* < 0.05; CBS vs PPA χ^2^ = 14.0, *p* < 0.001; and CBS vs bvFTD χ^2^ = 4.6, *p* < 0.05), and from onset (B: log-rank [Mantel-Cox] χ^2^ = 18.0, *df* = 3, *p* < 0.001; pairwise comparisons revealed significant differences for PSP vs PPA χ^2^ = 11.2, *p* = 0.001; PSP vs bvFTD χ^2^ = 4.8, *p* < 0.05; CBS vs PPA χ^2^ = 13.5, *p* < 0.001; CBS vs bvFTD χ^2^ = 5.7, *p* < 0.05). bvFTD = behavioral variant frontotemporal dementia; CBS = corticobasal syndrome; *df* = degrees of freedom; PiPPIN = Pick's Disease and Progressive Supranuclear Palsy Prevalence and Incidence; PPA = primary progressive aphasia; PSP = progressive supranuclear palsy.

Survival rates from PiPPIN assessment were significantly different depending on the diagnostic group (figure 2). Kaplan-Meier survival curves showed significant differences in survival from PiPPIN assessment (figure 2A; log-rank [Mantel-Cox] χ^2^ = 20.9, *df* = 3, *p* < 0.001; pairwise comparisons revealed significant differences for PSP vs PPA χ^2^ = 17.0, *p* < 0.001; PSP vs bvFTD χ^2^ = 6.0, *p* < 0.05; CBS vs PPA χ^2^ = 14.0, *p* < 0.001; and CBS vs bvFTD χ^2^ = 4.6, *p* < 0.05) and from onset (figure 2B; log-rank [Mantel-Cox] χ^2^ = 18.0, *df* = 3, *p* < 0.001, pairwise comparisons revealed significant differences for PSP vs PPA χ^2^ = 11.2, *p* = 0.001; PSP vs bvFTD χ^2^ = 4.8, *p* < 0.05; CBS vs PPA χ^2^ = 13.5, *p* < 0.001; CBS vs bvFTD χ^2^ = 5.7, *p* < 0.05). Date from onset was missing for 3 participants, hence the reduced sample size for figure 2B.

## Discussion

This study confirms the significance of apathy for survival, across the major syndromes associated with FTLD, including behavioral and language variants of FTD, PSP, and CBS. The results stem from a cross-sectional epidemiologic cohort that included community-based as well as specialist-center recruitment of prevalent cases, diagnosed according to current consensus diagnostic criteria. The carers' rating of apathy and functional decline (including self-care and motivation) was the most significant predictor of death within 30 months, even after adjusting for diagnostic group. The relevant apathy profile was weighted toward the Apathy Evaluation Scale, Neuropsychiatric Inventory, and Cambridge Behavioral Inventory subscores (principal component 2, table 3). The patients' age and general cognitive ability did not predict survival. In just 2.5 years in the cross-sectional cohort, 50 of 24 patients had died, in keeping with typical survival rates in previous studies of each disease. Despite differences in survival across groups (shortest for PSP, followed by CBS, bvFTD, and PPA), the diagnostic group did not predict death when apathy and other neurobehavioral profiles were included in the model.

These findings emphasize the prognostic importance of behavioral change in FTLD syndromes, over and above demographic features and diagnostic classification.^6,20,25^ This effect includes patients with diagnoses that are not defined by the presence of behavioral or personality change. One study classified patients with FTLD into specific phenotypes using latent profile analysis of neuropsychological, functional, and behavioral data.^26^ The prognosis was significantly worse in the “pseudomanic” group, who exhibited greater disinhibition and abnormal social conduct.^6^ It is of interest that their “pseudo-depressed” group had a better prognosis over time,^6^ despite the potential confusion between depression and apathy. Consistent with such studies, we suggest that apathy, but not depression, is most associated with poor outcomes and functional decline, reflecting the distinct underlying neurobiology of apathy and depression.^11^

Impulsive and challenging social behaviors (component 3) were not a significant predictor of reduced survival, although they may increase health care costs and carer burden. The importance of apathy, rather than disinhibition, in causing functional impairment and disability has been reported in bvFTD, perhaps because isolated disinhibition is unusual in bvFTD.^16^ Our study extends this result to other syndromes associated with FTLD.

The influence of behavioral changes on survival is further emphasized by the observation that patients with comorbid FTD-ALS (amyotrophic lateral sclerosis) survive up to a year less than patients with “motor only” symptoms. Apathy is common in ALS, affecting 40% to 80% of patients,^18^ and may precede motor symptoms. In the context of ALS, apathy is also an independent, negative prognostic factor, significantly predicting survival even after controlling for clinical factors and symptom duration: median survival of patients with ALS who have moderate to severe apathy was significantly shorter than those with mild apathy or no apathy (21.7 vs 49.9 vs 51.9 months, respectively).^18^

We have previously used voxel-based morphometry and diffusion tensor imaging to examine the structural brain changes associated with apathy and impulsivity, revealing marked white matter atrophy of the brainstem and widespread gray matter changes in thalamus, striatum, and cortical connections.^10,27^ Patients with PSP and CBS scored highly on symptoms associated with brainstem and midbrain atrophy. Prominent bulbar symptoms, reflecting brainstem pathology, increase the likelihood of death, in part by choking and aspiration.^28^ However, frontal atrophy has also been linked to poor outcomes in CBS,^20^ PSP, and FTD.^3^ Across FTLD, the “pseudomanic” phenotype classified in reference 6 (2009) with reduced survival demonstrated worse hypoperfusion of the frontal cortex. Worsening fronto-subcortical pathology, linked to behavioral changes, also predicts greater mortality in patients with autopsy-proven corticobasal degeneration and FTD.^9,20^

The lack of a significant influence of global cognitive status on survival is perhaps surprising, but accords with previous observations that behavioral change rather than cognitive decline is a marker of progression and prognosis in syndromes associated with FTLD.^6,25^ The relationship between apathy and cognition is complex: apathy might cause cognitive worsening but it may also be a consequence of cognitive decline. In predementia states, apathetic patients consistently show more rapid cognitive and functional decline and increased dementia conversion rates compared to nonapathetic groups.^17^ In Parkinson disease, apathetic patients converted to dementia more frequently than nonapathetic patients, in 18 months.^13^ Even in those who did not develop dementia, apathetic groups showed significantly greater cognitive decline, specifically in terms of executive function deficits in response inhibition and action initiation, emphasizing the link between cognition and apathy. In mild cognitive impairment and early Alzheimer disease, patients with apathy had an approximately 8-fold-greater risk of conversion to dementia over 3 years, even after controlling for potential confounds including age, sex, education, and episodic memory performance.^29^

This link between apathy, dementia, and survival, raises the hypothesis that successful symptomatic treatment may alter patient trajectories, including perhaps survival. Testing this hypothesis will require development of more effective symptomatic treatments targeting the underlying causes of apathy. Such an effect would challenge the long-standing dichotomy between symptomatic and disease-modifying therapies in FTLD.

The diagnostic group was not a significant predictor of survival in the presence of neurobehavioral profiles, despite significant differences in survival across groups if considered alone. This suggests that simple categorical diagnostic grouping (using clinical diagnostic criteria for the major syndromes associated with FTLD) is of limited value for prognostication.^6^ If observations are available that capture behavioral change in these disorders, such as apathy, then these are better able to predict survival than diagnosis alone. While neuropathologic studies have led to increased fractionation of FTLD syndromes, the influence of distinct pathologies on survival remain unclear, with both tau-positive and tau-negative cases correlating with reduced survival.^3,5,8,30,31^ Although variations in the survival rates across clinical phenotypes have been reported, in the presence of wide phenotypic variation, the diagnosis alone does not appear to be strongly predictive of survival.^6^

We argue instead that the presence and severity of apathy across the spectrum of FTLD disorders influences survival, while the remaining features that underlie diagnosis (but are not captured by the component) do not significantly influence prognosis. In other words, the differences in prognosis between syndromes (figure 2) are driven by the phenotypic features or their neurobiological correlates that are present across multiple disorders, albeit to a variable degree. This places the emphasis on the domains of impairment, akin to the Research Domain Criteria of neuropsychiatric disorders.^32^ In bvFTD, for example, current criteria require 3 of 6 core features. Therefore, 2 patients might have no clinical overlap, such that the presence of a clinical feature rather than the diagnosis per se determines prognosis. We also group the patients with PPA into a single group for analysis, in keeping with the high-level division of FTD into behavioral and language variants. This brings cases with marked clinical heterogeneity of svPPA, nonfluent variant PPA, and logopenic PPA into a single group. However, it has the advantage of approximating group sizes, while permitted phenotypic expression between subgroups to be retained in terms of the individuals' loading value on each of the neurobehavioral components.

The observation that the diagnosis category is not predictive of survival provides additional support for the transdiagnostic approach adopted by the PiPPIN study^2,10^ and has direct implications for the design of future clinical investigations. For a clinical trial of symptomatic treatments, we propose that emphasis should be placed on recruiting patients who present with that symptom (e.g., apathy) rather than on patients defined by a diagnostic label (e.g., bvFTD). In PiPPIN, apathy (component 2) was abnormal across the diagnostic groups (figure 1). Profound apathy and associated behavioral changes are increasingly recognized in PSP and CBS^33–37^ despite being largely overlooked because of predominant motor impairments.

Of note, it remains unclear whether the relationship between apathy and mortality is causal or merely correlational. Apathy may accelerate rapid decline to death or may represent a marker of other underlying factors that correlate with both apathy and survival, such as brainstem degeneration (the neural correlate of component 2^10^). Here, we do not have evidence of causality, but the relationship between apathy and survival raises the possibility that treating apathy would improve outcomes. Interventional studies are required, to either treat apathy at the behavioral level (symptomatic) or to target the underlying neural correlates (disease-modifying). However, it is likely that apathy in the context of syndromes associated with FTLD is multifaceted. For example, gray matter atrophy and white matter degeneration^10,27^ are accompanied by changes in noradrenaline, dopamine, and serotonin that modulate motivation, attention, and reinforcement learning.^38^ The quantification of these deficits, and the resulting neurobehavioral profiles such as apathy, may enable more effective stratification of patients for clinical trials.

A number of limitations should be acknowledged. Inherent to most clinical studies estimating survival in FTLD syndromes is the variability of clinicopathologic correlations, with potential misdiagnosis and thereby inaccurate within-syndrome estimates. Semantic dementia and PSP-Richardson syndrome have the highest accuracy (>90%), but for CBS, only approximately 60% have corticobasal degeneration.^39^ However, with the possible exception of logopenic PPA, those with a misdiagnosis often have another FTLD pathology, further emphasizing the benefits of a transdiagnostic approach. Here, we included collapsed diagnostic groups of PSP, CBS, bvFTD, and PPA in the logistic regression and acknowledge that the clinical diagnosis does not necessarily confirm the underlying cause of disease. We also note that logistic regression removes cases list-wise, reducing the power of the analysis. Because of the nature of neurodegenerative diseases, some patients were too severely impaired to complete all assessments. Missing variables resulted in the removal of 24 patients from the analysis, despite some recorded behavioral changes, demographics, and diagnosis. Although methods such as multiple imputation can be used to estimate scores based on other available measures, they are uncommon in logistic regression studies, and are not without assumptions that would be difficult to justify in our study (e.g., that missing data are missing at random). It is also possible that our cohort is biased or unrepresentative of the full spectrum of disorders associated with FTLD, although we sought to minimize such biases by the multiplicity of case ascertainment methods. We do not perform subanalyses by race, as >95% of the PiPPIN region patients are classed as “white Caucasians.” This prevents our examination of potential racial differences in disease expression and prognosis.

Apathy and related functional impairment in FTLD syndromes effectively predicts mortality. The prognostic importance of these neurobehavioral features may provide a means to effectively predict survival and stratify patients for clinical trials, for example into apathetic (rapid progressor) and nonapathetic (slow progressor) groups. Identification and enrollment of patients at greater risk of disease progression would maximize power to detect a therapeutic effect in forthcoming clinical trials. The prognostic importance of apathy highlights the need to develop more effective and targeted measurement tools to improve recognition and provide outcome measures for clinical studies. Indeed, effective symptomatic interventions targeting the neurobiology of apathy might even improve prognosis.

## Glossary

ALS: amyotrophic lateral sclerosis
bvFTD: behavioral variant frontotemporal dementia
CBS: corticobasal syndrome
*df*: degrees of freedom
FTD: frontotemporal dementia
FTLD: frontotemporal lobar degeneration
PiPPIN: Pick's Disease and Progressive Supranuclear Palsy Prevalence and Incidence
PPA: primary progressive aphasia
PSP: progressive supranuclear palsy
svPPA: semantic variant primary progressive aphasia
